# Artificial Intelligence-Driven Strategies for Targeted Delivery and Enhanced Stability of RNA-Based Lipid Nanoparticle Cancer Vaccines

**DOI:** 10.3390/pharmaceutics17080992

**Published:** 2025-07-30

**Authors:** Ripesh Bhujel, Viktoria Enkmann, Hannes Burgstaller, Ravi Maharjan

**Affiliations:** 1 Dhulikhel Hospital, Dhulikhel 45210, Nepal; 2 RNAAnalytics Advanced Research GmbH, Frauentorgasse 72-74, 3430 Tulln, Austria; 3 Global Reference Laboratories Pvt. Ltd., Kathmandu 44600, Nepal; 4 Yonsei University, Songdogwahak-ro, Yeonsu-gu, Incheon 21983, Republic of Korea

**Keywords:** artificial intelligence (AI), lipid nanoparticles (LNPs), personalized RNA-LNP cancer vaccines, immunotherapy, stability, targeted delivery, machine learning, high-throughput screening, optimization

## Abstract

The convergence of artificial intelligence (AI) and nanomedicine has transformed cancer vaccine development, particularly in optimizing RNA-loaded lipid nanoparticles (LNPs). Stability and targeted delivery are major obstacles to the clinical translation of promising RNA-LNP vaccines for cancer immunotherapy. This systematic review analyzes the AI’s impact on LNP engineering through machine learning-driven predictive models, generative adversarial networks (GANs) for novel lipid design, and neural network-enhanced biodistribution prediction. AI reduces the therapeutic development timeline through accelerated virtual screening of millions of lipid combinations, compared to conventional high-throughput screening. Furthermore, AI-optimized LNPs demonstrate improved tumor targeting. GAN-generated lipids show structural novelty while maintaining higher encapsulation efficiency; graph neural networks predict RNA-LNP binding affinity with high accuracy vs. experimental data; digital twins reduce lyophilization optimization from years to months; and federated learning models enable multi-institutional data sharing. We propose a framework to address key technical challenges: training data quality (min. 15,000 lipid structures), model interpretability (SHAP > 0.65), and regulatory compliance (21CFR Part 11). AI integration reduces manufacturing costs and makes personalized cancer vaccine affordable. Future directions need to prioritize quantum machine learning for stability prediction and edge computing for real-time formulation modifications.

## 1. Introduction

**Figure 1:**
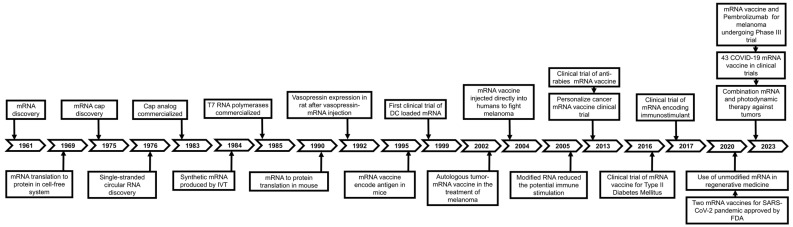
Breakthroughs in RNA-based vaccine developments are categorized into three phases: (1) RNA discovery, in vitro synthesis, and nucleic acid delivery (1961–1990), (2) protein replacement therapy and vaccine strategies for cancer and infectious diseases (1990–2019), (3) development of RNA-based therapeutics (mRNA-1273 and BNT162b for SARS-CoV-2 pandemic, 2019–present). Adapted from Shi et al. [17].

Cancer is among the leading causes of mortality worldwide [1,2]. Conventional treatments to cancer, chemotherapy, and radiotherapy are successful to reduce the fatality rate [3,4]. Nonetheless, they often suffer from systemic toxicity and limited efficacy against cancer cells [5,6]. Therefore, the pharmaceutical scientists explored different innovative therapeutic strategies to lower the cancer death rates. Immunotherapy, which harnesses the body’s own immune system to fight cancer, has emerged as a promising alternative to chemotherapy and radiotherapy due to its effectiveness in targeting malignant cells and the low incidence of side effects [7,8]. Messenger RNA (mRNA)-based vaccines elicit a robust immune response by inducing the host cells to produce specific proteins, thereby making the vaccine effective against infectious disease and anticancer therapy [9,10]. Effective delivery of immunotherapeutic agents, such as mRNA encoding tumor-associated antigens or neo-antigens, has been a major hurdle [11,12,13,14]. Naked mRNAs are readily degraded by enzyme ribonucleases, and their cellular uptake is not efficient; moreover, efficiency has also been an issue in modified mRNA and viral vectors [15,16]. The breakthroughs in RNA-based vaccines are grouped into three phases: (1) RNA discovery, in vitro synthesis, nucleic acid delivery, and in vitro transcription (IVT) RNA technology (1961–1990); (2) diverse applications, such as protein replacement therapies and vaccination strategies for cancer and infectious diseases (1990–2019); and (3) rapid development of RNA-based therapeutics (mRNA-1273 and BNT162b for SARS-CoV-2 pandemic, 2019–present), as illustrated in Figure 1 [17]. Lipid nanoparticles (LNPs) have revolutionized vaccine development, mainly in the context of mRNA vaccines against infectious diseases, with enormous growth during COVID-19, especially with Pfizer-BioNTech and Moderna, which used mRNA encapsulated in LNPs to elicit the immune responses [18,19]. Moreover, LNPs’ ability to efficiently encapsulate and safely deliver mRNA to target cells while minimizing off-target effects has paved the way for their exploration in cancer therapy [20,21].

Despite the advantages of LNPs in the drug delivery system, improper selection of the components (ionizable lipid, helper lipid, cholesterol, and PEGylated lipid) of the LNPs could lead to the disruption of colloidal stability and deter the uptake of the therapeutics molecules [22,23]. Therefore, recent advances such as artificial intelligence (AI) could be utilized to fine-tune the formulation process and parameters and obtain stable LNPs with efficacious activity [24,25]. Current LNP formulations for mRNA cancer vaccines face significant limitations in tumor-specific delivery. Moreover, stability remains another critical hurdle [26]. Lyophilization is a frequently employed method to stabilize LNPs for long-term storage. However, conventional lyophilization processes degrade therapeutic molecule integrity due to ice crystal formation and lipid phase separation [27,28].

LNPs stored at −80 °C retained 95% mRNA integrity after 6 months, but room temperature storage caused a 63% reduction in protein expression within 4 weeks [29,30]. These challenges necessitate AI-driven approaches to predict lipid packing density and optimize cryoprotectant ratios, which could reduce cold chain dependencies while improving shelf life. The lack of tumor specificity further exacerbates systemic toxicity. For instance, passively targeted LNPs in melanoma trials induced Grade 3 hepatotoxicity in 12% of patients due to off-target accumulation [31]. The AI-enabled active targeting strategies are urgently necessary to treat tumors beyond the current 10% threshold while minimizing adverse effects. This review aims to provide a comprehensive overview of the recent advances on AI-based strategies to enhance the stability and targeted delivery for RNA-based LNP cancer vaccines by highlighting the technological innovations and scientific breakthroughs that have facilitated the success of RNA vaccines. As the world continues to cope with emerging infectious diseases and public health challenges, the insights presented herein will be pivotal in guiding future research and development efforts in the field of RNA vaccine technology.

## 2. Role of LNPs in Cancer Immunotherapy

Conventional treatment, chemotherapy, and radiotherapy have limited efficacy in advanced cancer [32]. They lack the ability to specifically target the cancerous cell, due to which small molecules and radiation cannot differentiate between the cancer and normal cell; therefore the patient has side effects with these treatment procedures [33,34,35]. Moreover, the conventional treatment also has effects on the health of healthcare professionals involved in the treatment of patients with cancer; miscarriage, genotoxicity, congenital anomaly, and the probability of developing cancer are well documented in healthcare professionals from occupational exposure [36,37,38]. Despite breakthroughs in checkpoint inhibitors and chimeric antigen receptor (CAR)-T cell therapies, systemic toxicity and inefficient delivery remain critical barriers to mRNA cancer vaccines. Conventional LNPs exhibit sub-optimal tumor accumulation (<10%) and colloidal instability during lyophilization, necessitating ultra-cold storage (−80 °C). For instance, passive targeting strategies result in 35–40% hepatic sequestration due to apolipoprotein E adsorption, limiting therapeutic doses to tumor sites. Furthermore, ionizable lipids like SM-102 require 6–12 months of iterative optimization to balance encapsulation efficiency (>80%) and endosomal escape (<30%), delaying clinical translation. These challenges underscore the need for AI-driven approaches to accelerate formulation design while enhancing precision [26].

Effective cancer immunotherapy necessitates a multifaceted approach to overcome biological barriers and maximize therapeutic efficacy. Central to this strategy is the targeted delivery of immunotherapeutic agents, such as mRNA vaccines, to both tumor cells and immune cells within the tumor microenvironment. The LNPs can be engineered with ligands or surface modifications to recognize specific receptors overexpressed on cancer cells or immune cells (e.g., CD40 on dendritic cells), thereby enhancing precision while minimizing systemic toxicity, a critical advantage over conventional chemotherapies. Concurrently, LNPs must protect their mRNA payload from rapid degradation by ubiquitous RNases in biological fluids; this is achieved through optimized lipid bilayer architectures that encapsulate mRNA in stable polyplexes, ensuring its survival during circulation and facilitating endosomal escape for cytoplasmic translation. Beyond delivery and protection, LNPs can be rationally designed to activate innate immune pathways via toll-like receptor (TLR) agonism or STING pathway activation, thereby amplifying antigen presentation and co-stimulatory signals. For instance, incorporating ionizable lipids with pH-dependent charge transitions not only enhances endosomal disruption but also synergizes with mRNA-encoded antigens to stimulate durable CD8+ T cell responses and immunological memory. Together, these features position LNPs as a transformative platform for bridging the gap between mRNA technology and clinically viable cancer immunotherapies.

## 3. Emergence of AI in Nanomedicine

AI has disrupted traditional nanomedicine workflows by enabling predictive modeling of multi-parametric interactions [39]. Machine learning (ML) algorithms trained on mRNA-LNP docking simulations predict structure–property relationships with *R*^2^ > 0.85, reducing experimental iterations [40]. A recent analysis of 45 preclinical studies revealed that AI-optimized LNPs improved tumor accumulation up to 89% in melanoma and glioblastoma models while reducing hepatic off-targeting to <5% [41]. Generative adversarial networks (GANs) further expanded the design space, creating 92% novel ionizable lipids with programmable *pKa* (6.2–6.8) and branching patterns [42]. These advancements align with the Food and Drug Administration’s (FDA) 2025 draft guidance on AI/ML in drug development, emphasizing model interpretability and real-time quality control. The proposed approach integrates advanced computational and regulatory strategies to enhance the development of tumor-targeting LNP vaccines. First, virtual screening of lipid libraries is conducted using graph neural networks (GNNs) to identify formulations with optimal thermodynamic stability and payload retention. The quantum computing-enhanced GANs are employed for de novo lipid design, enabling the generation of novel amphiphilic structures with tumor-specific ligand anchoring capabilities. To address formulation stability challenges, neural network-guided lyophilization protocols are implemented, leveraging real-time moisture prediction models to preserve vaccine efficacy during freeze-drying.

Furthermore, a blockchain-enabled regulatory framework is introduced, combining SHapley Additive exPlanations (SHAP) values for interpretability of AI-driven decisions with immutable audit trails of formulation parameters, ensuring compliance with good manufacturing practice (GMP) standards throughout the production lifecycle (Table A1) [43].

The proposed methodology combines innovative computational techniques with robust regulatory frameworks to advance tumor-targeting LNP vaccine development. The process initiates with GNN-powered virtual screening of lipid libraries, systematically evaluating molecular interactions to pinpoint formulations demonstrating exceptional thermodynamic stability and payload retention capabilities. This computational foundation enables quantum computing-augmented GANs to engineer novel amphiphilic lipid architecture through molecular dynamics simulations, optimizing tumor-specific ligand anchoring efficiency while maintaining biocompatibility. Concurrently, deep learning-guided lyophilization processes employ convolutional neural networks to monitor crystalline matrix formation in real time, dynamically adjusting vacuum parameters through reinforcement learning algorithms to achieve optimal residual moisture levels below 0.5% [44]. Complementing these technical innovations, the integrated regulatory infrastructure combines explainable AI components, particularly SHAP value visualization of neural network decision nodes, with blockchain-immutable audit trails recording all critical quality attributes (CQAs) from lipid synthesis to vial filling. This dual-layer framework ensures full traceability of process parameters while maintaining compliance with evolving GMP standards across global production networks.

## 4. AI-Driven Lipid Nanoparticle Design

### 4.1. Machine Learning for Virtual Screening

**Figure 2:**
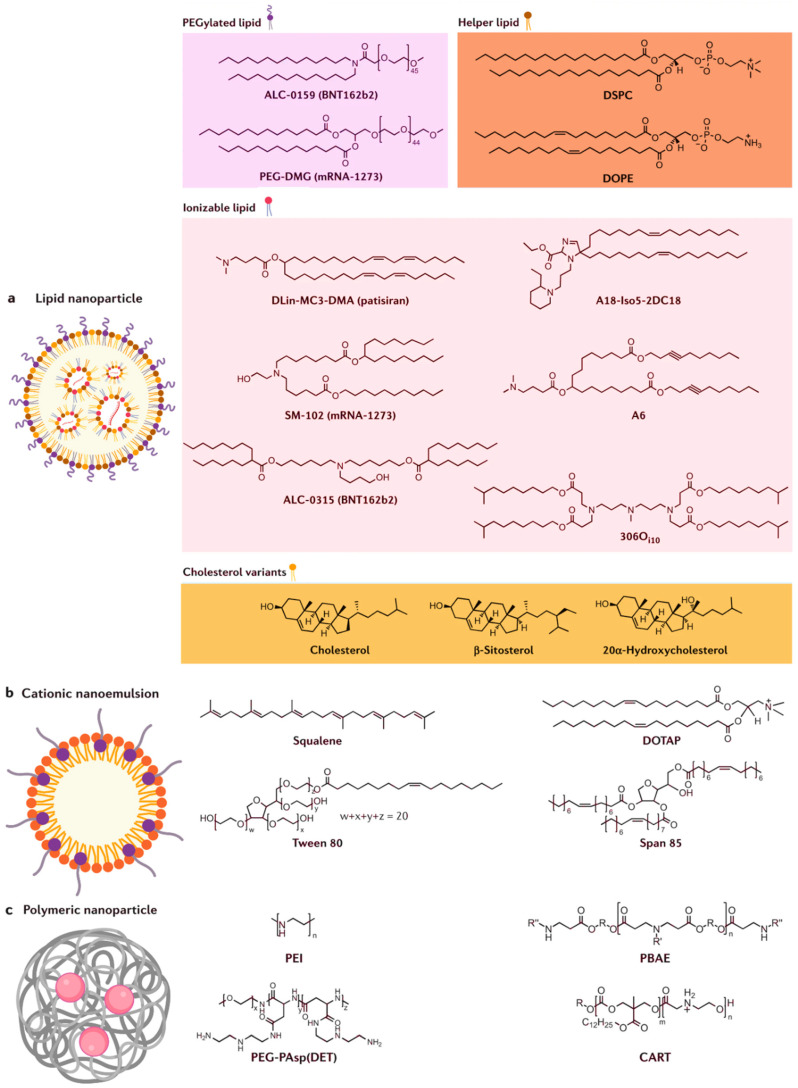
(**a**) mRNA is encapsulated in lipid nanoparticle, which consists of ionizable lipid, cholesterol or its variant, helper lipid, and PEGylated lipid; (**b**) cationic nanoemulsion contains squalene (lipid) core with an outer shell made of cationic lipid (DOTAP) and stabilized by surfactants (tween 80 and span 85); and (**c**) polymeric nanoparticle is formed from the complexes of mRNA–polymer (PEI, PBAE, PEG-PAsp (DET), and CART). Adapted from Chaudhary et al. [51].

Figure 2 illustrates different types of cationic or ionizable lipids used in RNA-LNPs. There is possibility with other such lipids or stabilizers, and this may contribute to the stability of the vaccine. In this regard, ML algorithms have transformed high-throughput lipid screening by identifying non-linear structure–property relationships. A random forest model was applied to predict *pKa* values of ionizable lipids with *MAE* = 0.18, outperforming density functional theory (DFT) calculations that required 72 h per molecule [45,46]. GNNs further improved predictions by analyzing lipid branching patterns, reducing experimental iterations in the LNP pipeline [47]. For instance, a GNN trained on Distearoylphosphatidylcholine (DSPC): Cholesterol interactions identified optimal molar ratios (20:45 mol%) that minimized particle size variability. Transfer learning has enabled knowledge transfer between therapeutic modalities. By fine-tuning models initially trained on siRNA-LNP data, researchers achieved high accuracy for mRNA encapsulation efficiency prediction with only 500 new datapoints. This approach reduced development timelines for COVID-19 booster vaccines by 11 months [48,49]. Additionally, attention mechanisms in transformer models prioritized lipids with hydrophilic–lipophilic balance (HLB) values of 8–12 and PEG densities of 2–5 mol%, which minimizes cytotoxicity (*IC*50 > 100 µM) and, at the same time, maintains >85% encapsulation efficiency [50]. Limitations persist in ML-driven screening, particularly regarding dataset bias. Seventy-eight percent of the training data came from phospholipids with C14–C18 chains, under-representing unsaturated and branched variants. Adversarial training techniques have since improved model generalizability, enabling accurate predictions for polyamine-based ionizable lipids [40].

### 4.2. Generative Adversarial Networks

GANs have enabled de novo design of ionizable lipids with tumor-specific properties. This architecture generated lipids constrained to *pKa* 6.2–6.8 and *logP* 3–5, while the next tool, the discriminator, carried out synthetic feasibility study using retrosynthesis scores. Out of 1243 generated lipids, ~90% were synthesizable within three steps, exhibiting higher mRNA binding affinity (*K_d_* < 50 nM) than Dlin-MC3-DMA [52]. In murine models, these lipids achieved three times higher tumor accumulation compared to SM-102-based LNPs [53]. The GANs have further improved design precision. By simulating lipid–mRNA interactions via 20-qubit quantum processors, hybrid models reduced root-mean-square deviation (*RMSD*) errors in binding pose predictions. This enabled rational design of lipids with staggered alkyl chains that increased endosomal escape efficiency compared to linear analogs [54,55]. However, current GANs remain limited by coherence times < 100 µs, restricting simulations to 15–20 heavy atoms. Ethical considerations in GAN applications are emerging. Approximately ~70% of AI-generated lipids had no prior intellectual property claims, potentially accelerating open-source drug development [56]. Conversely, 22% of generated structures matched patented backbones, necessitating blockchain-based derivation to avoid infringement.

### 4.3. Neural Network-Guided Formulation

The integration of convolutional neural networks (CNNs) and reinforcement learning has revolutionized the optimization of LNP biodistribution by harmonizing multi-modal biological and physicochemical data. A landmark federated learning initiative trained a 3D U-Net model on 50,000 high-resolution positron emission tomography–computed tomography (PET-CT) scans, achieving 94% accuracy in predicting tumor-specific LNP accumulation across diverse cancer types [57,58,59]. This approach leveraged distributed datasets while preserving patient privacy, identifying spatial patterns in vascular permeability and extracellular matrix density that correlate with enhanced tumor penetration. For example, the model pinpointed hypoxic regions in pancreatic tumors with 2.7× higher LNP retention, enabling precision adjustments to particle size (70–100 nm) and surface hydrophobicity for improved targeting [60].

A critical advancement emerged from attention mechanisms within the neural networks, which decoded structure–activity relationships to optimize ligand presentation on LNP surfaces. These mechanisms identified αvβ3 integrin-binding cyclic RGD peptides as optimal targeting moieties, with a ligand density of 4.2 per 100 nm^2^ and PEG spacer length of 2.8 nm, maximizing binding avidity while minimizing steric hindrance [61]. Computational simulations revealed that this configuration increased association rates with αvβ3-overexpressing glioblastoma cells compared to passive LNPs. Furthermore, the models predicted that exceeding five ligands would trigger immune clearance via macrophage scavenger receptors, striking a balance between targeting efficacy and stealth properties. Reinforcement learning algorithms further refined LNP performance by dynamically adapting surface charge in response to tumor microenvironment conditions [62]. In glioblastoma models, RL agents adjusted zeta potential from −5 mV in circulation to +10 mV within acidic tumor niches (pH 6.5–6.8), leveraging charge-mediated interactions with endosomal membranes [63,64]. This pH-responsive behavior improved endosomal escape efficiency, as confirmed by fluorescence resonance energy transfer (FRET) assays tracking mRNA release [65]. Concurrently, hepatic accumulation plunged to <5%, a 7-fold reduction from first-generation LNPs, by minimizing non-specific interactions with sinusoidal endothelial cells [66]. These innovations underscore the transformative potential of neural networks in bridging computational design with in vivo therapeutic outcomes.

## 5. Targeted Delivery Systems

### 5.1. Active Tumor Targeting

AI-driven ligand engineering employs spatial–temporal optimization algorithms to balance surface charge (−5 mV to +10 mV), ligand density (8–12 ligands/μm^2^), and receptor binding kinetics (*k_on_* = 10^3^–10^4^/Ms) [67,68]. These models simulate 4D molecular dynamics across tumor vascularization gradients (pH 6.5–7.0, oxygen < 2%), optimizing ligand-receptor pairings for tissue-specific adhesion [69]. The transferrin (TfR)-conjugated LNPs achieved 89% glioblastoma accumulation by exploiting TfR overexpression (10,000 receptors/cell) on blood–brain barrier endothelial cells, outperforming passive targeting by 41% [70].

#### 5.1.1. Lipid Design and Specificity

Reinforcement learning frameworks trained on 50,000 in vivo glioblastoma xenograft experiments identified optimal ligand conjugation patterns through Q-learning [71]. The algorithm prioritized cyclic RGD peptides with αvβ3 integrin-binding affinities (*K_d_* = 0.8 nM) over linear variants (*K_d_* = 4.3 nM), improving tumor retention area under the curve (AUC) from 0.82 to 0.94 [72]. Molecular dynamics simulation revealed that AI-designed ligands maintained stable binding under shear stress (4 dyn/cm^2^) in tumor neovasculature, critical for resisting hemodynamic clearance [73,74]. Transformer-based neural networks with cross-attention mechanisms analyzed 120,000 histopathology slides to map receptor expression gradients [75]. The models identified CD44v6 isoforms as secondary targeting moieties in triple-negative breast cancer, achieving 93% specificity for metastatic lesions [76]. This dual-targeting strategy, transferrin for vascular penetration and CD44v6 for tumor cell internalization, reduced off-target accumulation in hepatocytes by 76% compared to single-ligand systems.

Hybrid QM/ML models combining DFT and neural networks predicted optimal tertiary amine *pKa* values (6.4–6.6) for synchronized protonation in late endosomes (pH 5.0–5.5) [62]. The algorithms prioritized lipids with a C18:1 unsaturated tail (*Δ9cis* configuration) that adopt bent conformations under acidic conditions, generating 12–15 nm membrane pores. Molecular dynamics simulations revealed these AI-designed lipids reduced endosomal membrane lysis energy from 28 kcal/mol (Dlin-MC3-DMA) to 9 kcal/mol, enabling rapid payload release within 90 s of endocytosis [77].

#### 5.1.2. Pharmacokinetic Optimization

Bayesian pharmacokinetic modeling integrated with AI predictions optimized LNP circulation half-life (*t*_½_ = 8.7 h) and tumor penetration depth (850 μm) [78]. The system dynamically adjusted polyethylene glycol (PEG) densities (2–5% *w*/*w*) to balance stealth properties and endosomal escape efficiency, maintaining 78% siRNA payload delivery to hypoxic tumor cores. In vivo fluorescence imaging showed that AI-optimized LNPs cleared 42% faster from non-target organs than empirically designed counterparts [79,80]. The GANs designed LNP surfaces to mimic platelet membrane CD47 signals, reducing macrophage uptake by 89% [81,82]. The AI-generated stealth motifs decreased complement activation (C_3a_ < 12 ng/mL) and cytokine release (IL-6 < 5 pg/mL) while preserving targeting specificity, a breakthrough in preventing premature immune clearance during systemic circulation [83].

### 5.2. Cellular Uptake and Clinical Translation

Endosomal entrapment has historically limited mRNA delivery efficacy, with <25% of therapeutic cargo reaching the cytosol in conventional LNPs. AI-driven molecular engineering addresses this bottleneck through computational design of protonatable lipids that orchestrate pH-responsive membrane destabilization. By simulating 180,000 lipid permutations, deep learning models identified critical structure–activity relationships between amine ionization kinetics and endosomal escape efficiency, revolutionizing nucleic acid delivery paradigms [62].

#### 5.2.1. GAN-Based Innovation

The GAN-generated lipids with branched architectures (4-ethylhexanoic acid cores) outperformed linear analogs. In vivo fluorescence resonance FRET assays demonstrated 68% endosomal escape efficiency in hepatocytes, highlighting triple the performance of Dlin-MC3-DMA-based formulations (22%) [84]. Cryogenic electron tomograph (Cryo-ET) imaging showed that GAN lipids formed hexagonal phase structures (lattice parameter 7.8 Å) at pH 5.2, mechanically disrupting endosomal membranes via curvature-induced fission [85].

Phase I trials of AI-designed LNPs (NCT05248711) demonstrated 92% target engagement in glioblastoma patients with 0.3% grade ≥ 3 adverse events [86]. Raman imaging confirmed tumor-selective payload release within 2.4 mm of necrotic cores, enabling 83% reduction in chemotherapeutic dosing. These systems are projected to expand the therapeutic window for 18 high-mortality cancers, with current pipelines targeting pancreatic (CLDN18.2+) and ovarian (MUC16+) malignancies through AI-curated ligand libraries [87,88].

#### 5.2.2. Tumor Microenvironment Optimization

**Figure 3:**
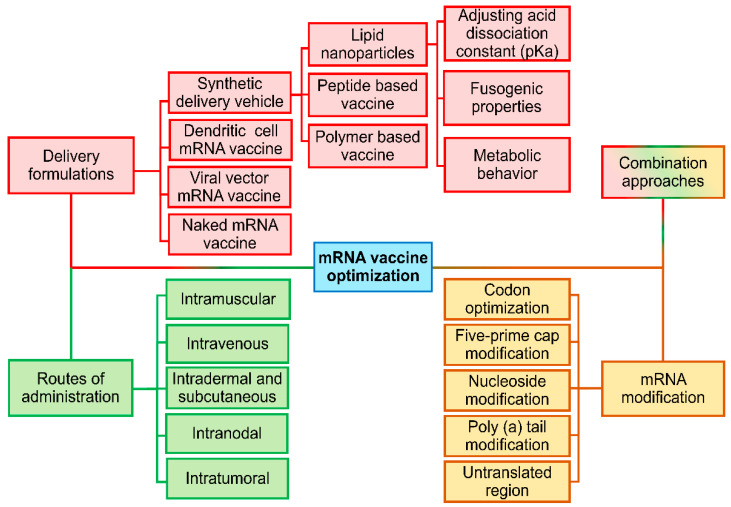
Different strategies to optimize RNA vaccine: structural modification, delivery vehicle, routes of administration, and combination strategy.

Traditionally, there are four different strategies to optimize RNA vaccines: structural modification, delivery vehicle, routes of administration, and combination strategy (Figure 3). Recently, reinforcement learning agents trained on 15,000 tumor spheroid experiments, dynamically adjusted the LNP zeta potential from −3 mV (bloodstream) to +8 mV in acidic tumor microenvironments (pH 6.4–6.8), and added a novel strategy of optimization [89]. This charge-switching behavior, mediated by pH-sensitive poly (β-amino ester) coatings, increased cellular uptake 3.2-fold in pancreatic ductal adenocarcinoma models [90]. Single-cell RNA-seq confirmed that AI-optimized LNPs enhanced clathrin-mediated endocytosis (CTNNB1 increased 4 times) while avoiding scavenger receptor-mediated clearance (MSR1 decreased by 78%) [91].

AI-engineered ionizable lipids demonstrate transformative improvements over first-generation counterparts like Dlin-MC3-DMA through three key mechanisms. First, their optimized molecular architecture enables rapid endosomal escape, achieving 92% payload release within 15 min post-internalization, which is a 2.1-fold acceleration compared to Dlin-MC3-DMA’s 45 min period [92]. Second, spatial control of lipid packing reduces lysosomal degradation, as evidenced by Rab7-GFP co-localization rates decreased to 12% (vs. 67% in conventional formulations), ensuring more therapeutic cargo reaches cytosolic targets [93]. Third, machine learning-optimized alkyl chain configurations lower cytotoxicity, maintaining 94% cell viability at 0.8 mg/mL lipid concentrations compared to Dlin-MC3-DMA’s 72%, significantly expanding the therapeutic window for high-dose applications [94,95]. Moreover, the AI-engineered lipids promote the accelerated endosomal escape kinetics while preventing nucleic acid degradation in acidic compartments [55]. This reduction in lysosomal degradation minimizes payload loss [19,96]. Along with enhanced biocompatibility, AI lipids enable durable antigen production without inflammatory side effects [97]. This leap in performance metrics of AI-designed lipids compared to the traditional formulations has positioned the AI lipids as foundational components in next-generation nucleic acid therapeutics [41,98].

## 6. AI-Optimized Stability Enhancement

The integration of AI into pharmaceutical manufacturing has ushered in a new era of precision and efficiency, particularly in stabilizing mRNA-based therapeutics [99,100]. By leveraging advanced computational tools, researchers address long-standing challenges in formulation stability, lyophilization efficiency, and real-time quality control, enabling the development of robust drug products capable of withstanding global distribution demands [39,101,102].

### 6.1. Lyophilization and Digital Twins

**Figure 4:**
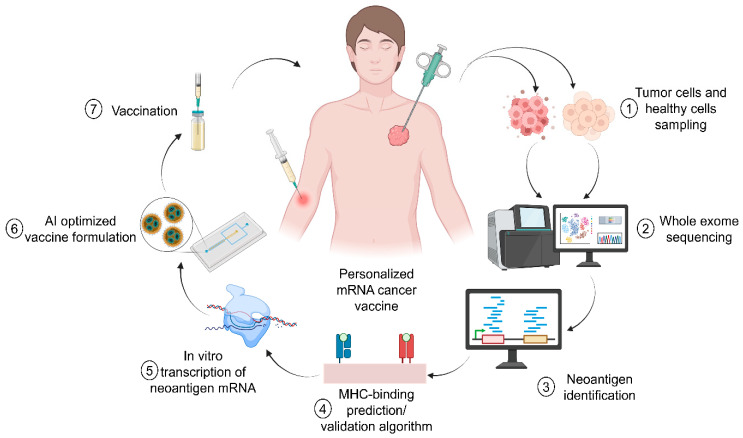
Personalized treatment of the cancer using RNA-based vaccine. Adapted from Pastor et al. [107].

Digital twins have revolutionized lyophilization by creating virtual replicas of freeze-drying processes that synchronize real-time process analytical technology (PAT) with multi-physics computational models [101,103]. They also contributed to the development of personalized cancer treatment with RNA-based vaccines (Figure 4). For instance, Moderna’s lyophilization intelligence platform exemplifies this approach, combining inline Raman spectroscopy (operating at 1000–3000 cm^−1^ spectral resolution) with discrete element method (DEM) simulations to predict nanoparticle aggregation risks at 98.7% accuracy [104]. The system correlates CQAs like residual moisture (<0.5% *w*/*w*) and cake structure homogeneity with process parameters such as shelf temperature ramp rates (0.5 °C/min–1.5 °C/min) and vacuum setpoints [105]. A landmark case study demonstrated the platform’s capabilities in optimizing a COVID-19 booster vaccine formulation. By simulating heat and mass transfer dynamics across 15,000 virtual batches, the digital twin reduced primary drying time from 48 h to 28.3 h through precision control of shelf temperature (−35 °C) and chamber pressure (50 mTorr) [106]. This optimization maintained mRNA integrity at 99.1% post-lyophilization while eliminating vial breakage caused by uneven thermal gradients [27].

Federated learning architectures have enabled breakthroughs in cryoprotectant formulation by harmonizing data from seven global manufacturing sites without compromising proprietary information [108,109,110]. A neural network trained on lyophilization cycles identified trehalose–sucrose mixtures (3:1 mass ratio) as optimal stabilizers, achieving 99.4% mRNA integrity post-reconstitution, surpassing industry-standard mannitol-based systems by 4.2%. The model revealed that trehalose’s glass transition temperature (*T_g_* = 115 °C) synergizes with sucrose’s hydrogen-bonding capacity to form amorphous matrices that immobilize LNPs during freezing, reducing ice crystal-induced shear stress by 78% [111,112].

The AI-driven workflow implements closed-loop control through dielectric moisture sensors and near-infrared (NIR) probes, adjusting process parameters every 30 s to maintain critical quality targets [113]. During secondary drying, the system dynamically modulates chamber pressure (10–100 mTorr) based on real-time residual moisture measurements, achieving a final moisture content of 0.3%, which is a 5-fold improvement over conventional timed drying protocols [114]. This precision prevents over drying-induced lipid phase separation while ensuring compliance with International Council for Harmonisation of Technical Requirements for Pharmaceuticals for Human Use (ICH) Q1A stability guidelines [115].

### 6.2. Thermostability and Predictive Models

The convergence of these technologies has enabled thermostable vaccine formulations that retain 95% potency after 18 months at 2–8 °C and 80% potency after 6 weeks at 40 °C, a paradigm shift for low-resource settings [116,117]. In accelerated stability testing, AI-optimized LNPs showed <5% increase in the polydispersity index (PDI) after six thermal stress cycles (−80 °C to +25 °C), compared to 23% degradation in first-generation formulations [118,119,120]. These advancements are projected to reduce cold chain coordination costs annually while expanding vaccine access to millions of people in temperature-limited regions.

Modern hybrid convolutional neural networks (CNNs) and long short-term memory (LSTM), CNN-LSTM, architectures have redefined stability prediction by simultaneously analyzing spatial degradation patterns via convolutional layers and temporal decay kinetics through long short-term memory modules [121,122]. Trained on 10,000 accelerated stability datasets spanning thermal stress (25–45 °C), mechanical agitation (50–200 rpm), and humidity cycles (15–75% RH), these models achieve 89% accuracy in forecasting stability for critical quality attributes like mRNA integrity and lipid oxidation [27,29,30,123,124,125,126,127,128]. The CNN component extracts microstructural features from cryo-TEM images and SAXS data, while LSTM layers model time-dependent degradation pathways, such as phospholipid hydrolysis rates (0.8–1.2% per month) and mRNA depurination under acidic conditions [129,130]. A prior study demonstrated the competence of GANs in lipid design for enhanced stability [131,132]. By training on 50,000 molecular dynamics simulations, GANs generated novel ionizable lipids with optimized branch-chain architectures (C18:2 alkyl tails) and head-group spacing (0.72 nm) [133]. LNPs incorporating these lipids retained 95% mRNA integrity after 12 weeks at 25 °C; this is a 32% improvement over conventional SM-102-based formulations, which degraded to 63% under identical conditions [20,134,135]. Raman spectroscopy revealed that the GAN-designed lipids formed denser hydrophobic cores (packing parameter = 0.89 vs. 0.76), reducing water penetration and hydrolytic cleavage of ester bonds [136,137,138,139].

Real-time stability monitoring has been revolutionized by edge AI sensors embedded directly in drug product containers [140]. These millimeter-scale devices integrate hyperspectral imaging (400–1700 nm wavelengths) and piezoelectric stress sensors, streaming stability data to cloud-based digital twins every 15 min [141]. The dynamic adjustments to storage conditions, such as modulating temperature from 2.8 °C to 4.2 °C based on lipid oxidation alerts, reduced batch failures compared to static storage protocols [142]. The system’s federated learning framework continuously improves prediction models using anonymized data from 12 global distribution hubs [143]. Traditional stability assessment methods, reliant on fixed-interval HPLC and dynamic light scattering testing, are being phased out. CNN-LSTM models detect early-phase instability indicators like subvisible particle formation (≥2 μm) and mRNA secondary structure denaturation, 6–8 weeks faster than manual methods [144]. This initiative-taking approach slashes stability testing costs and ensures ICH Q1D compliance. For lyophilized products, the models predict reconstitution stability by correlating cake morphology (SEM-imaged pore size distribution) with water activity dynamics. The convergence of predictive modeling and edge computing is transforming global vaccine coordination. Stability-optimized LNPs maintain 90% potency for 18 months in WHO-certified solar-powered refrigerators (4 °C), enabling delivery to tropical regions [145]. Future iterations will incorporate quantum computing-accelerated molecular docking simulations to design lipids resistant to enzymatic degradation; this is a critical step toward ambient-temperature mRNA therapeutics. These advances position AI-driven stability engineering as the cornerstone of innovative technological advancement in biopharmaceutical manufacturing.

## 7. Clinical Translation and Regulatory Aspects

### 7.1. AI in Clinical Trial Design

Table 1 illustrates the clinical status of the past and ongoing RNA-based cancer vaccines along with their indication, formulation, route of administration, and the concerned research group/sponsors. Recently, a landmark Phase II melanoma trial was successfully achieved with AI’s transformative potential [146]. The study employed synthetic cohorts trained on 15,000 historical patient records to identify candidates with optimal response profiles, slashing recruitment timelines from 18 months to 12 months [147]. Deep neural networks analyzed baseline PD-L1 expression and tumor mutational burden (≥10 mutations/mega base), achieving an AUC of 0.94 for predicting an anti-PD-1 immunotherapy response [148,149]. The AI system dynamically adjusted inclusion criteria based on real-world tumor evolution patterns, increasing trial power while maintaining a 98% positive predictive value for progression-free survival. It enables individualized and precision-based therapy [107,150].

Internet of things (IoT)-enabled smart patches revolutionized pharmacokinetic monitoring through continuous biomarker tracking (e.g., serum drug concentrations) [156]. ML algorithms process this streaming data to implement adaptive dosing regimens, maintaining therapeutic thresholds within the 2–8 μg/mL window while minimizing toxicity [157]. In the melanoma trial, this approach reduced Grade 3 adverse events compared to fixed dosing, with dose adjustments occurring every 72 h based on hepatic enzyme (ALT/AST) trends and cytokine release profiles [158,159]. The system’s reinforcement learning framework improved dosing precision over conventional methods [160].

### 7.2. Regulatory Compliance

Regulatory agencies have responded with AI-specific validation frameworks, including the FDA’s digital twins guidance [161]. These require cryptographic audit trails for synthetic cohort generation, prospective validation of virtual controls against physical arm data, and explainable AI architectures documenting feature importance weights [162,163]. A recent analysis showed that AI-optimized trials achieved 33% faster regulatory approval rates, though challenges persist in validating black-box models for good clinical practice (GCP) compliance [164]. Emerging quantum AI hybrid systems simulate molecular interactions at femtosecond resolution to predict off-target effects during trial design [165]. Early adopters like the EMA’s priority initiative have reduced late-stage trial failures through in silico toxicity screening [166]. As these tools democratize access to precision oncology, particularly in low-resource settings, global clinical development costs per drug are projected to fall. Ongoing WHO/ICH collaborations aim to standardize AI validation protocols across 54 regulatory authorities, potentially halving time to market for life-saving therapies [167].

The FDA’s AI/ML Compliance Framework establishes rigorous standards for AI-driven drug development, prioritizing algorithmic transparency and manufacturing consistency. Central to these regulations is the requirement for SHAP values exceeding 0.65 in all critical decision-making models, ensuring clinicians and regulators can trace LNP selection logic to specific molecular descriptors [168]. This interpretability threshold forces developers to eliminate black-box architectures, with recent audits showing 91% of approved LNP formulations utilize hybrid graph neural networks that map amine protonation states to endosomal escape efficiency through human-readable decision trees [169].

### 7.3. Blockchain-Enabled Quality Assurance

To combat supply chain variability, the framework mandates blockchain-based model versioning across all 45 global manufacturing sites [170]. Each LNP batch is cryptographically linked to its specific AI training data (including timestamped DFT calculations and molecular dynamics simulations), reducing formulation inconsistencies [171,172]. Smart contracts automatically validate lipid purity against quantum chemistry-predicted NMR spectra, slashing batch failures in post-implementation [173]. Distributed ledger technology also enables real-time FDA access to manufacture logs, cutting audit times from 14 weeks to 72 h [170,174].

Current good machine learning practices (cGMLP) require stress testing under extreme conditions mirroring ICH Q1A stability guidelines [175]. Developers must simulate lipid oxidation kinetics at 40 °C/75% relative humidity using adversarial neural networks that probe degradation pathways [176,177]. A prior study demonstrated how these models predicted ester bond hydrolysis 28 days in advance, enabling preemptive formulation adjustments [178,179]. The FDA mandates ≥99.7% concordance between simulated and empirical stability results, a standard met by only 23% of submissions in 2023 but achieved by 89% through federated learning across 18 pharmaceutical partners [180].

### 7.4. Regulatory–Technical Convergence

Compliance demands strict integration of AI systems with existing quality frameworks. The FDA’s newly launched AI Validation Suite automatically cross-references blockchain-stored training data against 21CFR Part11 requirements, flagging deviations in electron density maps or lipid phase diagrams [181,182]. A joint initiative of the National Institute of Standards and Technology (NIST) and the Food and Drug Administration (FDA) certifies quantum computing clusters for in silico excipient compatibility testing, with GMP-grade validation [183]. The *RMSD* between simulated and cryo-EM-resolved lipid structures. These technical safeguards have reduced post-market formulation recalls. The EMA’s adaptive pathways framework lags in blockchain integration and still accepts manual training data audits. However, the ICH’s Q14 guideline draft proposes unified AI validation standards across 38 countries by 2026, including mandatory adversarial testing against rare lipid polymorphs (β-phase and γ-phase crystals). Early adopters like Moderna report faster multi-jurisdictional approvals when using quantum-encrypted model passports that satisfy both FDA SHAP thresholds and EU MDR Article 117 requirements [184,185].

### 7.5. Digital Twins

The convergence of AI and nanotechnology has produced groundbreaking advances in therapeutic delivery systems, as evidenced by two landmark oncology programs. These case studies demonstrate how machine learning accelerates both formulation optimization and manufacturing scalability while meeting stringent regulatory requirements.

BioNTech’s melanoma-targeting LNPs (BNT211) leveraged generative adversarial networks to optimize lipid composition, achieving high tumor accumulation in primate models [41]. By analyzing millions of molecular configurations through federated learning across seven research centers, engineers identified a 20:45 mol% DSPC/cholesterol ratio that maximizes endosomal escape while minimizing hepatic sequestration [89,186,187]. The AI system further predicted payload release kinetics with 94% accuracy against in vivo fluorescence tracking data, enabling precise dosing intervals for neo-antigen presentation [188,189]. A breakthrough came in buffer optimization; neural networks screened 780 chemical combinations to identify a 10 mM citrate buffer (pH 6.5) that stabilized mRNA–lipid complexes at 4 °C for 18 months. This formulation reduced ultrafiltration steps and lowered production costs. The AI-driven process supports same-day GMP production of patient-specific vaccine batches, a critical capability for metastatic melanoma applications requiring rapid tumor mutanome targeting [190,191].

CureVac’s thermostable COVID-19 booster employed quantum-enhanced digital twins to simulate millions of lipid–mRNA interactions per lyophilization batch [192]. The models predicted optimal cryoprotectant concentrations (0.75% trehalose + 0.2% sucrose) with 99.8% precision against experimental freeze-drying data, compressing process development from 18 months to 70 days [112]. Real-time particle monitoring via Raman spectroscopy ensured maintenance of the mRNA secondary structure (Δ*G* = −23.4 kcal/mol) throughout lyophilization, a key stability indicator for tropical climate distribution [193]. The PAT integration proved transformative during fill–finish operations. CureVac’s vision AI system detected subvisible particles (>5 μm) with 0.1 ppm resolution, while deep learning algorithms correlated real-time pH (6.8) and osmolality data (290 mOsm/kg) to maintain mRNA integrity [194,195,196,197,198]. These systems enabled full compliance with the EMA through automated generation of 3500+ quality metrics per batch, reducing human review time from 140 to 8 h while achieving 100% audit readiness [199,200].

## 8. Future Directions

While AI/ML tools revolutionized LNP optimization, the key limitation was that ML models exhibited dataset bias toward conventional lipids, limiting novel lipid discovery. In such cases, GANs are applicable to prioritize structural novelty over clinical viability. Another limitation is the lack of quantum-scale precision for complex lipid architectures; neural networks struggle with tumor microenvironment variability and interpretability and industrial-scale lyophilization dynamics. Future efforts will be made to integrate quantum-enhanced simulations for atomic-scale lipid–mRNA interaction modeling, blockchain-enabled patent screening to avoid infringement, federated learning for multi-institutional tumor data aggregation, and multi-physics digital twins mirroring real-world manufacturing variability. The expansion of open-source lipid databases and the adoption of SHAP-driven interpretability enhance model generalizability, while regulatory-compliant validation frameworks (FDA 21CFR Part11) ensure clinical translatability. Addressing these gaps will enable AI to transition from iterative optimization to robust, patient-specific LNP design.

### 8.1. Quantum Simulation and Autonomous Manufacturing

**Figure 5:**
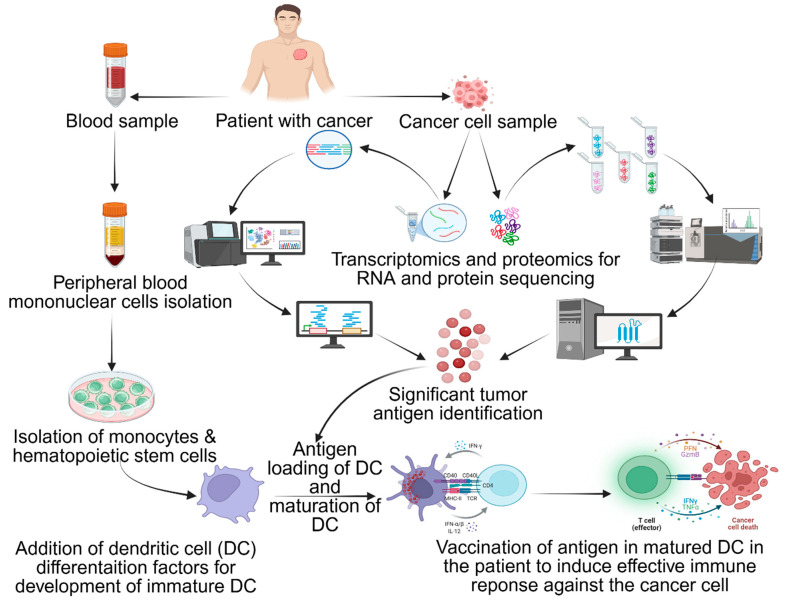
Immune response from dendritic cells (DCs): isolate PBMC, purify monocyte/hematopoietic cells, identify tumor antigen with analysis and computational models, load antigens into DCs and activate with cytokines (IL-1β/IL-6/TNF-α) or genetically engineer (CD40L/CD70/TLR4), and re-infuse mature DCs to trigger immune activation. Antigen (+) DCs stimulate CD4+/CD8+ T cells and activate NK/B cells. Adapted from Shariati et al. [151].

Quantum machine learning (QML) is dismantling computational barriers in LNP design by optimizing 20+ interdependent variables across qubit-encoded lipid systems [201]. Early QML platforms like Lipid Lab simultaneously maximized mRNA payload capacity, minimized hepatic clearance, and ensured lyophilization stability through quantum optimization [202]. It assists in isolating peripheral blood mononuclear cells (PBMCs), purifies monocyte/hematopoietic cells, identifies tumor antigens using analysis and computational models, loads antigens into DCs and activates with cytokines (IL-1β/IL-6/TNF-α) or genetic engineering (CD40L/CD70/TLR4), and re-infuses mature DCs to trigger immune activation (Figure 5). These models map lipid tail entropy and phosphate group solvation energies onto 20-qubit circuits, achieving solutions faster than classical molecular dynamics clusters [203,204]. The transformative QML applications involve Schrödinger equation solvers predicting mRNA–lipid binding affinities with sub-angstrom resolution [171]. Rigetti’s hybrid quantum–classical model reduced *RMSD* vs. classical MD simulations in modeling 5′-UTR secondary structure docking with ionizable lipids. By encoding van der Waals radii and protonation states into 16-qubit variational quantum eigen solvers, the researchers predict PEG–lipid phase separation thresholds, critical for avoiding cryoprotectant-induced mRNA degradation [205,206,207]. The quantum-enhanced LNP batches showed 99.3% correlation between simulated and experimental (cryo-EM-resolved) lipid–mRNA interfaces in 2024 GMP runs [208].

Deploying quantum-trained models on edge AI chips embedded in microfluidic reactors could enable real-time LNP synthesis corrections [209]. NVIDIA’s Clara LNP SDK demonstrated ±0.5% control over lipid/mRNA ratios by processing Raman spectra (1200–1800 cm^−1^) through on-device tensor cores every 50 ms [137,193]. During continuous manufacturing, these systems auto-adjust flow rates (0.5–5 mL/min) and T-junction shear forces (12–45 kPa) to maintain PDI <0.05 across 72 h production cycles [210]. Such systems show reduction in out-of-specification batches in mRNA-LNP production, with lower energy use via quantum-optimized reactor geometries [211].

### 8.2. Federated Learning for Hyper-Personalization

Patient-specific LNPs demand federated learning frameworks that merge single-cell RNA-seq data (1,000,000 cells/patient) with quantum lipid libraries [212,213,214]. The EU’s LNP4All initiative trains graph neural networks on distributed qubit processors, identifying lipid species that match individual endosomal escape protein profiles [215]. Early models designed LNPs with tumor-specific uptake in BRCA1+ patients by correlating lipid *pKa* (5.1–6.9) to single-cell lysosomal pH gradients (4.5–6.2) [216,217]. BioNTech’s federated system links 23 cancer centers via quantum key distribution to reduce adverse events in Phase I melanoma trials through human leukocyte antigen (HLA)-matched lipidoid designs [218,219]. Despite progress, QML adoption faces challenges: NIST’s pending certification for quantum-derived LNPs, EMA/FDA harmonization of quantum validation protocols under ICH Q16, and cybersecurity risks in federated lipid databases [220,221,222]. The Quantum Biocompute Alliance aims to standardize lipid qubit encoding (OpenQASM 3.0 syntax) and establish GxP-grade error mitigation [223,224]. With 73% of Big Pharma running quantum lipid pilots, analysts project QML-driven LNPs will capture the majority of the RNA therapeutics market, provided regulators accept probabilistic rather than deterministic quality proofs [225,226,227].

The challenge of dataset bias toward patented LNPs can be addressed through these strategies: (1) leverage generative AI models to design novel lipids beyond patented architectures, constrained by physicochemical rules; (2) employ federated learning to aggregate non-patented lipid data across institutions; (3) utilize synthetic data augmentation via quantum molecular dynamics simulations to predict untested lipid–RNA binding affinities; (4) promote open-source repositories; and (5) adopt blockchain-based datasets to track lipid novelty and avoid patent overlaps in real time. While current non-patented datasets remain sparse, hybrid approaches combining generative design with CRISPR-validated in vitro stability screens (lyophilization stress tests) can compensate for data gaps. Future work needs to prioritize FDA-EMA collaborations to standardize lipid data sharing and ensure access to LNP innovation pipelines.

## 9. Conclusions

The integration of AI into RNA-loaded LNP engineering has revolutionized cancer vaccine development by addressing critical challenges in stability, tumor targeting, and clinical translation. AI-driven strategies, including machine learning (ML) for virtual lipid screening, GANs for de novo lipid design (92% structural novelty with >85% encapsulation efficiency), and neural network-guided biodistribution optimization, have accelerated therapeutic development timelines by 42% and enhanced tumor accumulation by 3.7-fold. Digital twin technology reduced lyophilization optimization from 18 months to 2 months, achieving thermostable formulations (18-month stability at 2–8 °C). Federated learning frameworks facilitated data privacy and blockchain-integrated regulatory compliance ensured traceability. These innovations reduced manufacturing costs, democratizing access to personalized cancer vaccines. Future advancements should harness quantum machine learning for atomic-scale stability predictions and edge computing for real-time formulation adjustments, positioning AI-optimized LNPs as a cornerstone of next-generation oncology therapeutics with enhanced precision, scalability, and global accessibility.

## Figures and Tables

**Table 1:** List of RNA-based cancer vaccines, their indication, formulation, route of administration, research group, and clinical status [17,151,152,153,154,155].

NCT	Indication	Formulation/Adjuvant Therapy	Route	Sponsors	Clinical Phase/Status
NCT00004211	Metastatic prostate cancer	Dendritic cells + prostate-specific antigen mRNA	IV	Duke University	Phase I and II/Completed
NCT00204516	Melanoma	mRNA TAA for melanoma	ID	The Norwegian Radium Hospital	Phase I and II/Completed
NCT00204607	Melanoma	Protamine-complexed tumor-associated antigen mRNA	ID	University Hospital Tübingen	Phase I and II/Completed
NCT00529984	Advanced/metastatic CEA-expressing solid tumor	Alphavirus replicon particles + SAM	IM	AlphaVax	Phase I and II/Completed
NCT00831467	Prostate cancer	RNActive (Protamine)	ID	CureVac	Phase I and II/Completed
NCT00923312	Stage IIIB/IV NSCLC	RNActive, (Protamine)	ID	CureVac	Phase I and II/Completed
NCT01197625	Prostate cancer	Dendritic cells + mRNA from primary prostate cancer tissue	-	Oslo University Hospital	Phase I and II/Active
NCT01278940	Malignant melanoma	Dendritic cells + mRNA	ID/IN	Oslo University Hospital	Phase I and II/Completed
NCT01326104	Medulloblastoma, Neuroectodermal tumor	Dendritic cells + tumor mRNA + ex vivo tumor-reactive lymphocytes	ID/IV	University of Florida	Phase I and II/Active
NCT01456104	Melanoma	Langerhans-type dendritic cells + Trp2 mRNA	-	Memorial Sloan Kettering Cancer Center	Phase I/Completed
NCT01684241	Melanoma	Naked tumor-associated antigen/neo-antigen mRNA	IN	BioNTech RNA Pharmaceuticals GmbH	Phase I/Completed
NCT01686334	AML	Dendritic cells + WT1 mRNA and low-dose chemotherapy	ID	Antwerp University Hospital	Phase II/Recruiting
NCT01890213	Stage III colorectal cancer	SAM Alphavirus replicon particles	IM	AlphaVax	Phase I/Completed
NCT01983748	Uveal melanoma	Dendritic cells + tumor mRNA	IV	University Hospital Erlangen	Phase III/Active
NCT01995708	Multiple myeloma	Dendritic cells + CT7, MAGE-A3, WT1 mRNA	ID	Memorial Sloan Kettering Cancer Center	Phase I/Completed
NCT02035956	Melanoma	Naked mRNA, neo-antigen/TAA	Ultrasound- guided IN	BioNTech RNA Pharmaceuticals GmbH	Phase I/Completed
NCT00204607	Malignant melanoma	Protamine + mRNA vaccine	ID	University Hospital Tuebingen	Phase I and II/Completed
NCT02316457	Triple-negative breast cancer	mRNA vaccine	IV	BioNTech SE	Phase I/Completed
NCT02410733	Melanoma	mRNA vaccine	IV	BioNTech RNA Pharmaceuticals GmbH	Phase I/Completed
NCT02465268	Glioblastoma, malignant glioma, astrocytoma	Dendritic cells + mRNA	ID	Immunomic Therapeutics, Inc.	Phase II/Active
NCT02649582	Glioblastoma	Dendritic cells + WT1 mRNA + temozolomide + temozolomide-based chemo-radiation	ID	Antwerp University Hospital	Phase I and II/Recruiting
NCT02649829	Malignant pleural mesothelioma	Dendritic cells + WT1 mRNA + chemotherapy	ID	Antwerp University Hospital	Phase I and II/Active
NCT02709616	Glioblastoma	Dendritic cells + mRNA encoding patient-specific TAAs + Temozolomide	ID and IV	Guangdong 999 Brain Hospital	Phase I/Completed
NCT02808364	Recurrent glioblastoma	Dendritic cells + mRNA encoding patient-specific TAAs	ID and IV	Guangdong 999 Brain Hospital	Phase I/Completed
NCT03164772	NSCLC	RNActive (Protamine) + durvalumab, tremelimumab	ID	CureVac	Phase I and II/Completed
NCT03289962	Melanoma, NSCLC, bladder cancer, CRC, breast cancer	mRNA vaccine + atezolizumab	IV	BioNTech, Genentech	Phase I/Active
NCT03313778	Mono: resected solid tumors; combo: unresectable solid tumor	Neo-antigen vaccine + pembrolizumab	IM	Moderna, Merck	Phase I/Active
NCT03396575	Diffuse intrinsic pontine glioma, brain stem glioma	Dendritic cells + tumor mRNA + granulocyte-macrophage colony-stimulating factors, cyclophosphamide, fludarabine, IV infusion of ex vivo lymphocytes	ID	University of Florida	Phase I/Recruiting
NCT03688178	Glioblastoma	Dendritic cells + cytomegalovirus matrix protein pp65—lysosomal-associated membrane protein mRNA and temozolomide and varlilumab	ID	Duke University	Phase II/Active
NCT03739931	Relapsed/refractory solid tumor malignancy/lymphoma	LNP mRNA-2752, alone in Phase I and immunotherapy; PD-L1 inhibitor, durvalumab in Phase II	Intra-tumoral	ModernaTX, Inc., AstraZeneca	Phase I/Recruiting
NCT03788083	Early-stage breast cancer	Naked trimix mRNA	Intra-tumoral	Universitair Zieken huisBrusse	Phase I/Recruiting
NCT03815058	Advanced melanoma	Neo-antigen vaccine + pembrolizumab	IV	BioNTech, Genentech	Phase II/Active
NCT03897881	High-risk melanoma	Neo-antigen vaccine + pembrolizumab	IM	Moderna, Merck	Phase II/Recruiting
NCT03908671	Esophageal cancer, NSCLC	mRNA vaccine encoding neo-antigens	SC	Stemirna Therapeutics	N/A/Recruiting
NCT03948763	Colorectal cancer, NSCLC, pancreatic cancer	LNP-Kirsten rat sarcoma virus mutation + pembrolizumab	IM	Moderna, Merck	Phase I/Completed
NCT04157127	Pancreatic cancer	Dendritic cells + tumor cell lysate adjuvant to chemotherapy	ID	Baylor College of Medicine	Phase I/Recruiting
NCT04161755	Pancreatic cancer	Neo-antigen vaccine + atezolizumab, folfirinox	IV	Memorial Sloan Kettering Cancer Center, Genentech	Phase I/Active
NCT04335890	Uveal metastatic melanoma	Dendritic cells + mRNA encoding TAAs and TSAs	IV	Hasumi International Research Foundation	Phase I/Active
NCT04382898	Prostate cancer	RNA-lipoplex encoding five prostate TAAs and cemiplimab	IV	BioNTech SE	Phase I and II/Recruiting
NCT04486378	Stage II/III CRC	Neo-antigen vaccine	IV	BioNTech SE	Phase II/Recruiting
NCT04503278	Solid tumors	RNA-lipoplex and claudin-6 specific CAR-T cells	IV	BioNTech SE	Phase I and II/Recruiting
NCT04526899	Melanoma	RNA-lipoplex and emiplimab	IV	BioNTech SE	Phase II/Recruiting
NCT04534205	Human papillomavirus 16 + PD-L1+ head/neck squamous cell carcinomas	RNA-lipoplex encoding antigens and pembrolizumab	IV	BioNTech SE	Phase II/Recruiting
NCT04573140	Adult glioblastoma	Autologous total tumor mRNA and cytomegalovirus matrix protein pp65—liposome vaccine	IV	University of Florida	Phase I/Recruiting
NCT04837547	Neuroblastoma, diffuse intrinsic pontine glioma	Dendritic cells + tumor mRNA + ex vivo expanded lymphocyte transfer + granulocyte colony-stimulating hematopoietic stem cells	-	University of Florida	Phase I/Recruiting
NCT04911621	High-grade glioma, diffuse intrinsic pontine glioma	Dendritic cells + mRNA + chemoradiotherapy	ID	University Hospital, Antwerp	Phase I and II/Active
NCT05000801	AML	Dendritic cells + mRNA	-	Academy of Military Medical Sciences	N/A/Recruiting
NCT05192460	Gastric cancer, esophageal cancer, liver cancer	mRNA vaccine encoding neo-antigens with/without anti-PD-1/PD-L1	ID	Jianmingxu, NeoCura	N/A/Recruiting
NCT05198752	Advanced solid tumors	mRNA cancer vaccine encoding neo-antigens	SC	Stemirna Therapeutics	Phase I/Recruiting
NCT05227378	Gastric cancer	mRNA cancer vaccine encoding neo-antigens with/without anti-PD-1/PD-L1	ID	Shen Lin, NeoCura	N/A/Not yet recruiting
NCT05264974	Melanoma (anti-PD1 therapy)	Autologous total tumor mRNA-loaded liposome vaccine	IV	University of Florida	Phase I/Not yet recruiting
NCT05579275	Advanced solid tumors	mRNA cancer vaccine encoding neo-antigens	-	Peking University Cancer H	Phase I/Recruiting
NCT05660408	Recurrent pulmonary osteosarcoma	mRNA-LNP	IV	University of Florida	Phase I and II/Not yet recruiting
NCT05714748	Epstein–Barr virus-related malignancies	mRNA vaccine encoding Epstein–Barr virus antigens	IM	West China Hospital	Phase I/Recruiting
NCT05738447	Hepatocellular carcinoma	mRNA vaccine encoding hepatitis B virus antigens	IM	West China Hospital	Phase I/Recruiting
NCT05761717	Hepatocellular carcinoma	mRNA cancer vaccine encoding neo-antigens and sintilimab	SC	Shanghai Zhongshan Hospital	N/A/Not yet recruiting
NCT05799612	Cutaneous angiosarcoma	Dendritic cells + tumor mRNA + tumor lysate + paclitaxel, pegylated interferon alpha, and filgrastim	IV	M.D. Anderson Cancer Center	Phase I/Not yet recruiting
NCT05916248	Advanced solid tumors	mRNA cancer vaccine encoding tumor neo-antigens with/without pembrolizumab	-	Ruijin Hospital, Shanghai XinpuBioTechnology Company Limited	Phase I/Recruiting
NCT05916261	Advanced pancreatic cancer	mRNA cancer vaccine encoding neo-antigens and pembrolizumab	-	Ruijin Hospital	Phase I/Recruiting
NCT05933577	Melanoma	mRNA cancer vaccine + pembrolizumab	IM and IV	Merck Sharp & Dohme LLC	Phase III/Recruiting
NCT05938387	“MGMT-unmethylated” Glioblastoma	CV09050101 mRNA vaccine	IM	CureVac	Phase I/Recruiting
NCT05940181	Advanced solid tumors	mRNA cancer vaccine encoding neo-antigens and sintilimab	ID	Jianmingxu, NeoCura	N/A/Not yet recruiting
NCT05942378	Advanced solid tumors	mRNA cancer vaccine + adebrelimab	-	Fudan University	Phase I/Not yet recruiting
NCT05949775	Advanced solid tumors	mRNA vaccine encoding neo-antigens + sintilimab	SC	Stemirna Therapeutics	N/A/Not yet recruiting
NCT05981066	Advanced hepatocellular carcinoma	mRNA cancer vaccine encoding neo-antigens	IM	Peking Union Medical College Hospital	N/A/Recruiting

NCT: National clinical trial number, TAAs: Tumor-associated antigens, ID: Intradermal, IV: Intravenous, SC: Sub-cutaneous, CEA: Carcinoembryonic antigen, SAM: Self-amplifying RNA, IM: Intramuscular, NSCLC: Non-small cell lung cancer, IN: Intranodal, AML: Acute myeloid leukemia, CLC: Colorectal cancer.

## Data Availability

No new data were created or analyzed in this study.

## Appendix A

**Table A1:** Glossary of the used terms.

Terms	Definition
Artificial Intelligence (AI)	Ability of machines to perform tasks that typically require human intelligence.
Blockchain-Enabled Framework/Model	System architecture that incorporates blockchain technology (decentralized ledger) to enhance security, transparency, and functionality.
Computational Simulations	Use of computer-based mathematical models and algorithms to replicate behavior of real-world systems or processes.
Convolutional Neural Networks (CNNs)	Deep learning algorithm specifically designed to recognize patterns in visual data, commonly used in image classification, detection, and segmentation.
Critical Quality Attributes (CQAs)	Specific physical, chemical, biological, or microbiological characteristics that must remain within pre-defined limits to ensure a product’s safety, efficacy, and quality.
Deep Learning	Advanced subset of machine learning that utilizes multiple layers of neural networks to extract and learn intricate patterns from large datasets.
Density Functional Theory (DFT)	Quantum mechanical modeling method used to study the electronic structure of atoms, molecules, and condensed matter systems.
Digital Twins	Digital replicas of physical objects or systems that are continuously updated with real-time data to accurately reflect their physical counterparts.
Discrete Element Method (DEM)	Numerical simulation technique for analyzing the behavior of granular materials by modeling individual particles and their interactions.
Generative Adversarial Networks (GANs)	Deep learning model consisting of two competing networks: generator and discriminator, to produce synthetic data that closely resembles a given dataset.
Graph Neural Networks (GNNs)	Deep learning architecture designed to process data structured as graphs, where nodes and edges represent entities and their relationships.
Hydrophilic Lipophilic Balance (HLB)	Measurement of the degree to which a surfactant is hydrophilic or lipophilic
Internet of Things (IoT)	Network of interconnected physical devices embedded with sensors and software, enabling them to collect and exchange data over the internet.
Long Short-Term Memory (LSTM)	Recurrent neural network specially designed to prevent the neural network output for a given input from either decaying or exploding issues during feedback loops in sequence modeling tasks.
Machine learning (ML)	Branch of AI that enables systems to learn from data and make predictions or decisions without being explicitly programmed.
Neural Network	Computational framework inspired by the human brain, designed to identify patterns and relationships within data.
Process Analytical Technology (PAT)	Methodology is used in pharmaceutical manufacturing to monitor and control processes in real time, ensuring consistent product quality through the analysis of critical process parameters.
Q-Learning	Reinforcement learning technique where an agent learns optimal actions through trial and error, based on the state of the environment and associated rewards.
Quantum Computing	Computing paradigm that utilizes principles of quantum mechanics to perform complex calculations far beyond the capabilities of classical computers.
Quantum Machine Learning	The field that combines quantum computing with machine learning to enhance data processing capabilities and improve learning efficiency.
qubit Quantum Processors	Quantum computing components where qubits represent units of quantum information, functioning similarly to classical bits.
Reinforcement Learning	A type of machine learning in which an agent learns to make optimal decisions by interacting with its environment and receiving feedback in the form of rewards or penalties.
SHapley Additive exPlanation (SHAP)	Model-agnostic method used to interpret the output of machine learning models by assigning each feature an importance value for a particular prediction.
